# Lip creams with propolis special extract GH 2002 0.5% versus aciclovir 5.0% for herpes labialis (vesicular stage): Randomized, controlled double-blind study

**DOI:** 10.1007/s10354-018-0667-6

**Published:** 2018-11-07

**Authors:** Jagienka Jautová, Hana Zelenková, Katarína Drotarová, Alena Nejdková, Božena Grünwaldová, Marie Hladiková

**Affiliations:** 1 Department of Dermatology, Medical University of Kosice, Trieda SNP 1, 04011 Kosice, Slovakia; 2 Dermatological Clinic DOST, Dr. Pribulu 2, 08901 Svidnik, Slovakia; 3 Dermatology Department, First Private Hospital, Lucna 57, 04015 Kosice, Slovakia; 4 Policlinica II Dermatology Ambulance, C. Spanyola 43, 01001 Zilina, Slovakia; 5 Dermatology Ambulance, Bernolakova 2476, 95501 Topolcany, Slovakia; 6 Department of Medical Informatics, Second Facultyof Medicine, Charles University of Prague, V Úvalu 84, 15006 Prague, Czech Republic

**Keywords:** Propolis special extract, Herpes labialis, Lip sores, Aciclovir, Propolis-Spezialextrakt, Herpes labialis, Lippenherpes, Aciclovir

## Abstract

A lip cream with special propolis extract GH 2002 at a concentration of 0.5% (199 patients) was tested against aciclovir 5% (198 patients) in the treatment of episodes of herpes labialis under double-blind conditions. Upon inclusion, all patients were in the vesicular phase. Application was five times daily of approximately 0.2 g of cream to the entire upper and lower lip. The primary parameter was the difference in time between groups to complete encrustation or epithelization of the lesions. Secondary endpoints were the course of typical herpes symptoms (pain, burning and itching, tension and swelling), the global assessment of efficacy and the safety of application. The predefined clinical situation was reached after a (median) 3 days with propolis and 4 days with aciclovir (*p* < 0.0001). Significant differences in favor of propolis were also found for all secondary parameters. No allergic reactions, local irritations or other adverse events occurred.

## Zusammenfassung

Unter Doppelblindbedingungen wurde eine Lippencreme mit 0,5 % Propolis-Spezialextrakt GH 2002 (199 Patienten) gegen eine Creme mit 5 % Aciclovir (198 Patienten) bei der Behandlung von Episoden des Herpes labialis getestet. Bei Einschluss waren alle Patienten in der vesikulären Phase. Die Anwendung der Creme erfolgte 5‑mal täglich mit je etwa 0,2 g auf die gesamte Ober- und Unterlippe. Als primärer Endpunkt war der Gruppenunterschied in der Zeit bis zur vollständigen Verkrustung oder Epithelisierung der Läsionen definiert. Sekundäre Endpunkte waren der Verlauf der typischen Herpessymptome (Schmerzen, Brennen und Jucken, Spannung und Schwellung), die Gesamtbewertung der Wirksamkeit und die Anwendungssicherheit. Die vordefinierte klinische Situation wurde unter Propolis nach (im Median) 3 Tagen und mit Aciclovir nach 4 Tagen erreicht (*p* < 0,0001). Signifikante Unterschiede zugunsten von Propolis zeigten sich auch bei allen sekundären Endpunkten. Es traten weder allergische Reaktionen noch lokale Irritationen oder andere Nebenwirkungen auf.

## Introduction

Herpes labialis as a cutaneously manifested viral disease is caused by the herpes simplex virus type 1 (HSV-1), but also by the herpes simplex virus type 2 (HSV-2). The infection manifests itself primarily in the area of the lips, but can also broaden into a herpes oticus or herpes ophthalmicus. The vast majority of the population is a carrier of the herpes viruses, which persist in the ganglia of the spinal cord [1, 2]. Due to infectious diseases, stress, solar radiation or immunodeficiency—particularly in old age—the latently present herpes viruses become active and cause the infection process. The course is well-known. The first prodromal symptoms are tingling and itching. They develop into a papular, erythematous phase, which then turns into a vesicular phase with virus-containing vesicles, and then into an open erosive phase followed by incrustation and healing.

The classical local therapy for herpes labialis is carried out with virostatics such as aciclovir or penciclovir. With substances of the aciclovir type, whose effect is exclusively based on an antiviral activity [3–7], we found that a truly satisfactory local therapy of the debilitating and painful herpes labialis is not ensured [8, 9]. Active substances that, apart from the antiviral properties, also show antibacterial, antiphlogistic and, if possible, also local anesthetizing effects could be advantageous in the management of herpes labialis.

We became aware of such an active substance: a special extract preparation of propolis (GH 2002). This natural substance has been shown to possess potent antiviral effects *in vitro* in models of herpes simplex virus I and II [10, 11], and it also showed antibacterial effects in low concentrations in vitro [12]. Propolis is a resinous material collected by honeybees from the buds and bark of various trees and plants [13]. Propolis has been used in traditional medicine for at least two thousand years [14]. Chemically, propolis is a complex composition of substances, which may vary according to its geographic origin [14–17]. The most biologically important and prevalent constituents appear to be phenolic acids, flavonoids, terpenes, cinnamic acid and caffeic acid [18, 19].

Propolis is associated with a broad spectrum of biological activities, among them antioxidant, anti-inflammatory, antibiotic, antiviral, antifungal and antitumoral properties [15, 20–28].

Dermatological studies [8, 29] performed with the special propolis extract GH 2002 (with pollen, waxes and resins removed to reduce the risk of allergic reactions) confirm the in vitro results and verified that significant results were achieved in the treatment of herpes labialis, applying 0.5% of this active substance in a cream. Recently, a single-blind study in 379 herpes labialis patients showed significant therapeutic results in favor of a cream with 0.5% GH 2002 compared with aciclovir cream 5%, both for the primary parameter incrustation/healing and for the secondary parameters pain, itching, swelling and tension [9].

In all previous studies with the specific propolis extract GH 2002 the patients were included when they were in the prodromal or the papular/erythematous phase of the herpes episode. It was still unknown whether the observed benefits of propolis lip cream would still be observed when treatment was stated at a later phase of the herpes episode, i. e., the vesicular stage. The present study was therefore designed to include only patients with manifest blisters. It was highly important that the inclusion criterion of the stage of the herpes episode was respected: The study design was created with the aim of a comparison with published data with the application of aciclovir.

## Materials and methods

### Study design

The objective of this clinical trial was to compare a lip cream with propolis special extract GH 2002 0.5% with a cream containing aciclovir 5%, with patients included in the vesicular phase of the episode. The study was designed as a randomized double-blind, parallel group, reference-controlled multicenter trial (clinical phase III). It was performed at the Dermatological Department of the Medicinal Faculty of the University SK Kosice and at four dermatological out-patient departments and clinics.

### Ethical considerations

The study was registered under EudraCT No. 2012-004372-19 and performed in accordance with the Declaration of Helsinki/Edinburgh and followed ICH-GCP (International Convention on Harmonization – Good Clinical Practice) guidelines. Agreement from all ethics committees presiding over the trial centers was obtained. The acceptance of the relevant state authorities (SUKL in Bratislava [Slovakian Drug Authorization Authority]) was also obtained. Written consent was obtained from all patients after information.

### Blinding

The study sponsor prepared a fully blinded random list with the aid of a random number generator (RandList v1.2, Heise Medien GmbH, Hannover, Germany) in blocks of ten. The random list distributed the patients to the two study arms in a balanced manner. It was closed after labelling and was only re-opened after the study and the database were officially closed, and the statistical analysis was performed. The random code was not available to the physicians. During the trial the random numbers were allocated to the patients in the sequence of their inclusion.

### Study medications

Patients with typical herpes labialis eruptions (vesicular) were recruited and randomized to one of two treatment groups: propolis special extract GH 2002 cream 0.5% or aciclovir cream 5%.

The active substance propolis special extract GH 2002 is obtained from the natural product propolis, which comes from a defined bee pasture in Central Europe. Raw propolis is purified in a special procedure and freed from the accompanying substances like wax, resins and pollen. This purification leads to an extract enriched with flavonoids, polyphenols and phenyl carboxylic acids with clinically demonstrated antiviral effects [9, 30]. One study arm was given a lip cream containing 0.5% of the propolis extract (developmental medicinal product manufactured by Gehrlicher Pharmazeutische Extrakte, 82547 Eurasburg, Germany; cream batch No. 35/0116; extract batch No. 9803)—for the composition see [9].

The reference arm applied a cream with 5% aciclovir (reference, original cream manufactured by STADA Arzneimittel AG, Bad Vilbel, Germany; original batch number 212). Excipients were cetyl alcohol, dimethicone, glyceromacrogol-250-monostearate, liquid paraffin, propylene glycol, soft white paraffin and purified water. For the assurance of blinding this cream was further treated by Gehrlicher Pharmazeutische Extrakte under laminar flow conditions to add small amounts of sugar colorant and honey flavor to mimic the color, smell and taste of propolis special extract GH 2002 (batch No. 212-51/0317). The manufacture of both creams was fully documented.

Both creams were filled into neutral tubes with 10 ml of preparation each, and the tubes were labelled with the pre-prepared random code. The creams were undistinguishable with respect to appearance and consistency. This procedure assured full blinding of physicians and patients.

### Dosing and study duration

Patients were told to apply the dispensed cream five times daily (every 3–5 h) to the complete upper and lower lip. The single dose corresponded to approximately 0.2 g, corresponding to a daily exposure to approximately 1 g of cream.

Clinical examinations took place on day 0 (inclusion), day 1 or 2, and days 3, 4 and 5. For patients still showing symptoms with a requirement of prolonged treatment on day 5, additional examinations were planned for day 7 ± 1 and day 9 or 10.

### Study parameters

The primary parameter to objectify the development of the herpes labialis stage was the time until complete encrustation or epithelization of the lesions was reached. The typical symptoms of herpes (pain, burning/itching, tension/swelling) were documented as secondary parameters, as was the global assessment of efficacy and skin tolerance.

Special emphasis was given to the exact description of the stage of herpes labialis episode.

### Inclusion and exclusion parameters

Inclusion was possible for patients of both sexes at the age of 18–80 years showing visible eruptions (vesicular stage) having lasted no more than 30 h prior to inclusion. As an additional condition patients had to report a history of at least four previous episodes of herpes labialis.

This study focused on the effects of propolis extract when first applied in the vesicular phase. Patients presenting with symptoms of the prodromal stage, i. e. with no further symptoms than burning or tension of the lips, were not eligible. Likewise, patients with symptoms progressed beyond the vesicular phase, such as papules, erythema, erosions or encrustations, could not be included. Other local or systemic antiviral or antibacterial therapies or treatments with corticosteroids were not allowed. Hypersensitivity to any component of the study preparations, concomitant viral infections, acquired or malignant immunodeficiency including HIV or leukemia, the severity of the herpes labialis requiring systemic treatment, or the concurrent use of other topical preparations or systemic antiviral medication were additional criteria for exclusion from study participation. Additional antiviral medications were also not permitted during the trial.

### Case number calculation

The results of a previously performed dose-finding study [29] were used for the case number calculation. In the dose-finding study in patients with herpes labialis, the dose group with 0.5% propolis extract had reduced the time to complete encrustation by approximately one day when compared to a 0.1% concentration. The latter was still active to some extent. The expectation for this study was therefore that with a concentration of 0.5% propolis in the cream the time period to complete encrustation or epithelization should be around 1.5 days shorter than an untreated episode. In placebo comparisons, aciclovir has been found to reduce the duration of herpes labialis episodes by approximately one day. Based on these findings, it was assumed for the purpose of case number calculation that there should be an advantage of propolis over aciclovir of approximately 0.5 days in the comparison of median time to full encrustation or epithelization. Further assumptions for the case number calculation were a drop-out rate of 20% and a power of at least 80% in the superiority testing. As a result, 190 patients had to be included in each group.

### Statistics and study parameters

The statistical software was IBM SPSS Statistics version 21.0.0 (IBM-SPSS Inc., Armonk, NY).

The intention to treat (ITT) group was defined as the population of patients exposed to the trial medication and having returned for at least one control examination. The per protocol group (PP) included all patients compliant with the protocol, and for whom clinical data from the visits at day 0 through day 5 was available. The PP data set also includes the patients who terminated study participation before day 5 because they had already reached the stage of full encrustation or epithelialization.

The visits to assess the development of the therapy were made on day 1 or 2, day 3, day 4 and day 5, with additional examinations on day 7 ± 1 and day 9 or 10 if the therapy was not previously successful.

The primary study parameter was the study time in days until lesions were completely encrusted or epithelized (study start to first day when all vesicles or the erosive phase/open wound had disappeared). As in the previous study, where propolis was already applied at an earlier stage of the herpes episode, the superiority testing was based on the transformation of time differences until full encrustation or full epithelialization in the two groups. The statistical comparison was made by comparing the time in days until 50% of the population of the groups had reached the defined endpoint of the study [9].

The first statistical approach was a non-inferiority testing, to be followed by superiority testing if non-inferiority was found. As in the previous study [9], superiority calculation was made in the ITT population by means of a one-sided Mann–Whitney U test, using the SPSS Exact module giving exact *p* values. The level of significance set at 0.025. The threshold for superiority was defined as a difference between medians of the time for the groups to reach full encrustation or epithelization with a minimum of 0.5 days in favor of propolis.

Secondary parameters were the development of typical burdensome symptoms of herpes labialis (i. e., pain, itching and burning, and tension and swelling), the physician’s global assessment of efficacy, and the assessment of safety of application. The *p*-values calculated for secondary endpoints did not possess a confirmatory value. Compliance testing was made visually by inspection of tubes brought back by the patient to each visit.

Pain was rated on a 100 mm visual analogue scale (VAS). Individual improvement of pain was calculated from the average pain at days 2 and 3 minus the pain at inclusion. The Mann–Whitney U test was used for intergroup comparisons at a two-sided significance level of 0.05. Itching/burning and tension/swelling were rated on a four-step verbal rating scale, with 0 = absent, 1 = mild, 2 = moderate and 3 = severe. The global rating of efficacy was also made on a four-step verbal rating scale, with 0 = poor, 1 = moderate, 2 = good and 3 = very good. The Fisher’s exact test served for intergroup comparisons with respect to the presence of the symptoms itching and burning as well as tension/swelling, and for the statistical evaluation of the physician’s global assessment of efficacy. Differences of symptom severity were addressed using the Mann–Whitney U test at a two-sided significance level of 0.05. Tolerance and safety of application were to be examined descriptively. In the case of the observation of adverse events, it was foreseen to apply the Mann–Whitney U test or the Fisher’s exact test for intergroup significance testing, as applicable.

Furthermore, it was foreseen to replace missing values for the primary and secondary parameters using the worst case imputation method. This method replaces missing values in the propolis group by the worst value of the propolis group, and missing values in the aciclovir group by the best value of the aciclovir group. The worst case imputation method favors the comparator and thus increases the robustness of superiority test results.

## Results

**Fig. 1:**
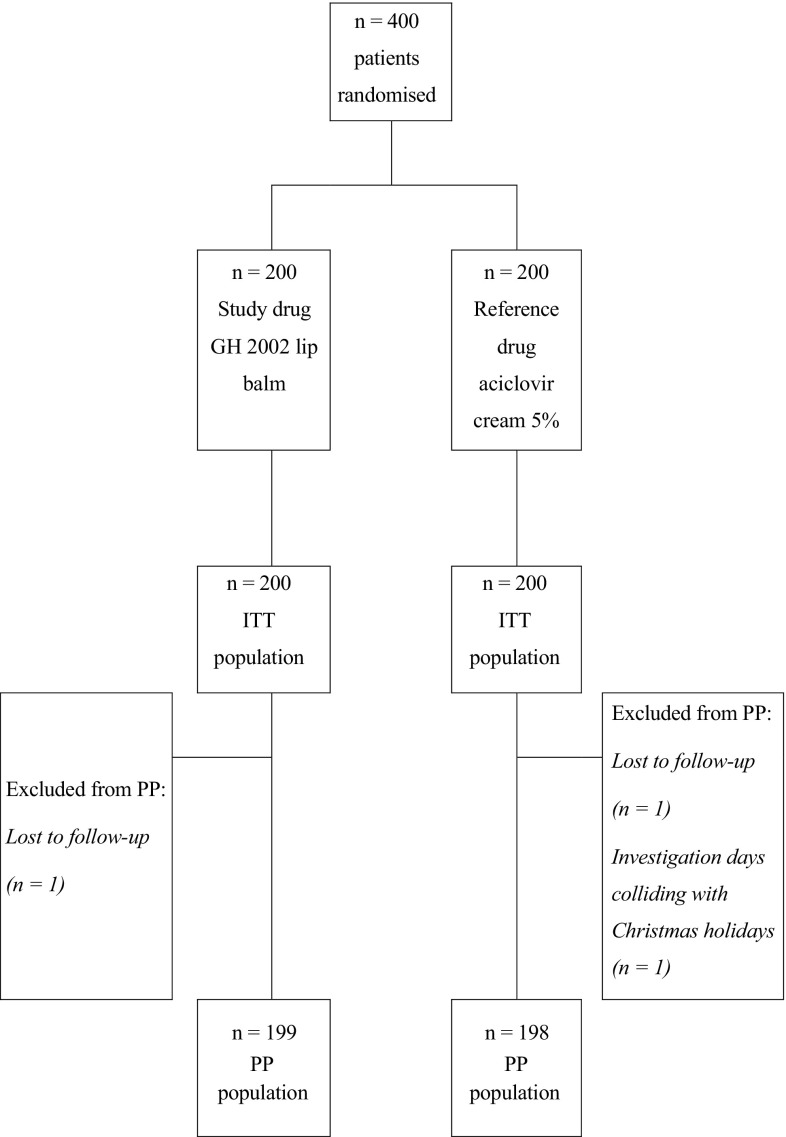
Flow chart of patient recruitment. *ITT* Intention to treat, *PP* per protocol

Four hundred patients were prepared, included and equally distributed to the two study groups (200 patients each, ITT population). Both study groups were well comparable for age and gender (Table 1) as well as for the typical symptoms of a herpes episode: there was no statistically significant difference between groups. There was one early study termination with propolis and two with aciclovir (Fig. 1). The PP population therefore consisted of 199 and 198 patients in the propolis and the aciclovir group.

**Table 1:** Demographic data

	Propolis group (*n* = 200)	Aciclovir group (*n* = 200)
*Gender*		
Female	75%	79%
Male	25%	21%
*Age (years)*	41.2 ± 15.4	42.1 ± 15.4

Compliance was rated inconspicuous in all cases after visual inspection of the medication tubes returned to each visit.

### Superiority testing: time to complete encrustation or epithelialization

**Fig. 2:**
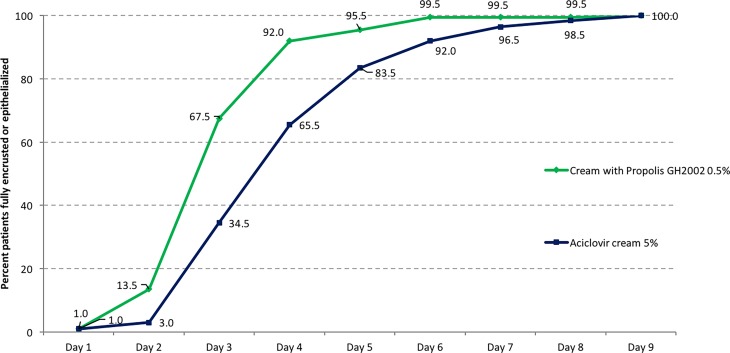
Cumulative percentage of patients reaching full encrustation or epithelialization in the course of the study (intention to treat [ITT] population, *n* = 200 per group)

**Fig. 3:**
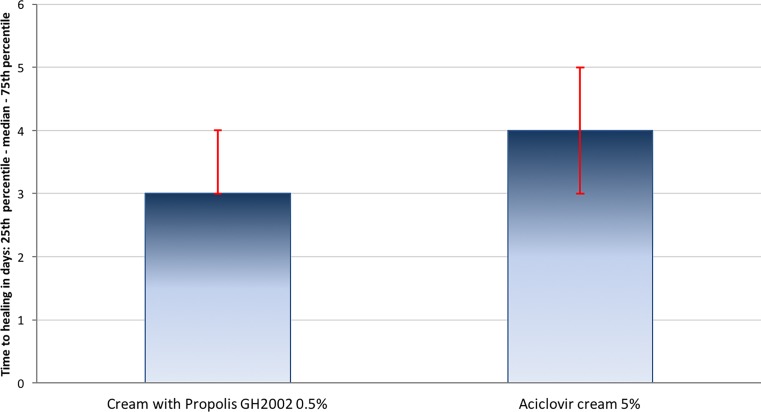
Superiority testing: time to complete encrustation or epithelialization (intention to treat [ITT] population)

Statistical testing resulted in the demonstration of non-inferiority of propolis versus aciclovir in both, the ITT and the PP population (*p* < 10^−7^, data not shown), hence superiority testing could be performed. Full encrustation or epithelialization was reached earlier under treatment with propolis than with aciclovir (Fig. 2). The mean time to full encrustation or epithelialization in 50% of the group population was 3.32 ± 1.00 days in the propolis group (median: 3 days) and 4.26 ± 1.43 days in the aciclovir group (median: 4 days). Propolis treatment was confirmed superior over aciclovir treatment (ITT, *p* < 0.0001; Fig. 3).

### Development of pain

**Fig. 4:**
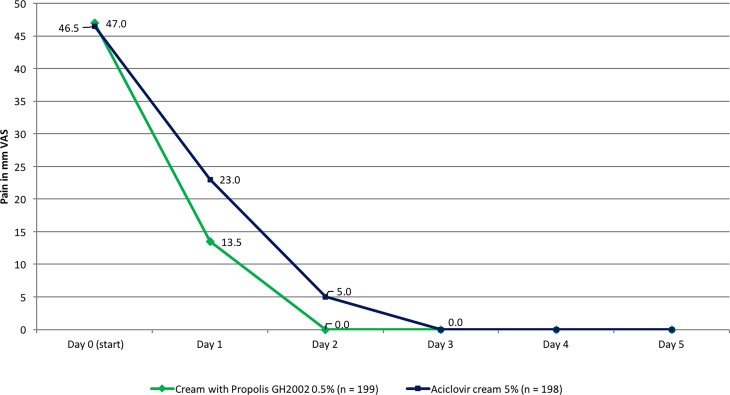
Development of pain in the course of the study (per protocol [PP] population). *VAS* Visual analogue scale

Pain assessments by the 100 mm VAS scale showed a significant pain reduction especially between day 0 and day 2, with a distinct advantage for propolis treatment demonstrated by reaching a full remission from pain one day earlier than with aciclovir treatment (Fig. 4). This advantage was quantified by calculating the difference of the mean pain on treatment days 2 and 3 versus baseline: with propolis, a reduction of the mean VAS score by 34.2 ± 23.3 points was reached, with aciclovir the reduction was 28.0 ± 22.3 points (PP population; the values for the ITT population were practically identical). The difference between groups was statistically significant (PP and ITT population, *p* < 0.01).

### Itching/burning and tension/swelling

**Fig. 5:**
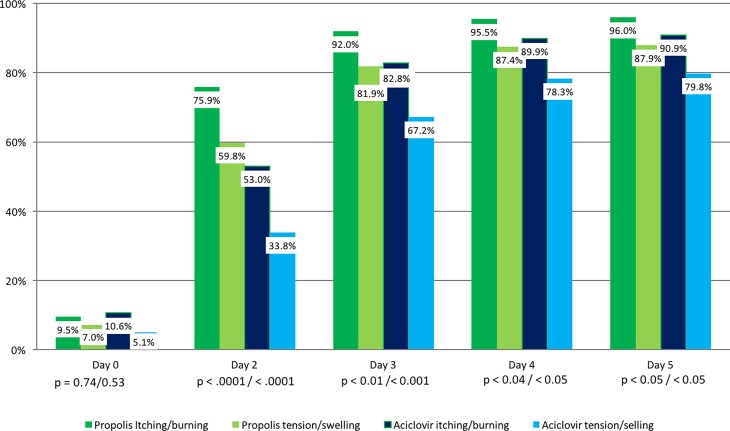
Percentage of patients free of itching/burning and tension/swelling in the course of the study (per protocol [PP] population; Fisher’s exact test)

The presence of itching/burning and tension/swelling as well as the severity of symptoms decreased continuously over the study period in both groups, but more quickly with propolis. In all cases a statistically significant group difference was observed on all study days from day 2 to day 5. Table 2 presents the development of symptom severity, whereas Fig. 5 presents the percentage of patients where the symptom was absent, both in the PP population. There was no different outcome when the calculation was made with the ITT population.

**Table 2:** Frequency of the symptoms itching/burning and tension/swelling in the course of the study (Mann–Whitney test)

	Propolis	Aciclovir	Propolis	Aciclovir	Propolis	Aciclovir
	Day 0	Day 0	Day 2	Day 2	Day 5	Day 5
*Itching/burning*						
Absent (%)	9.5	10.5	76.0	53.0	96.0	91.0
Mild (%)	23.5	26.5	21.0	33.0	4.0	4.0
Moderate (%)	48.5	40.5	2.0	14.0	0	5.0
Severe (%)	18.5	22.5	1.0	0	0	0
–	*p* = 0.99		*p* < 0.00001		*p* < 0.05	
*Tension/swelling*						
Absent (%)	7.0	5.5	60.0	34.0	88.0	79.5
Mild (%)	17.5	20.5	33.5	46.5	11.5	16.5
Moderate (%)	50.5	46.0	6.0	19.5	0.5	4.0
Severe (%)	25.0	28.5	0.5	0.0	0.0	0.0
–	*p* = 0.55 (n. s.)		*p* < 0.00001		*p* < 0.05

Effects set in more quickly in the propolis-treated group than in the aciclovir group: On Day 2, tension and swelling was absent in 119 patients of the propolis group (59.8%), whereas the same condition was reached in the aciclovir group by 67 patients (33.8%).

### Global assessment of efficacy

**Fig. 6:**
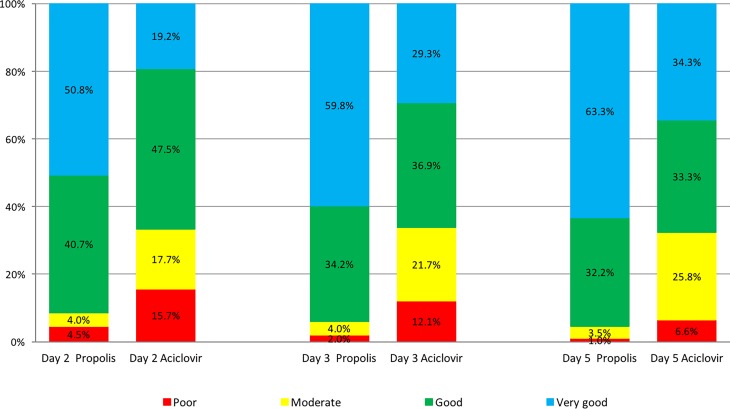
Global assessment of efficacy by the physician (per protocol [PP] population)

The global assessment of efficacy was rated on study days 2, 3 and 5. In all cases a statistical comparison between study groups was made (Mann–Whitney test), resulting in each single comparison in a highly significantly better outcome for propolis (PP population, *p* < 0.00001; Fig. 6). There was no different result when the calculation was made for the ITT population.

### Safety of application

There were no adverse effects during this study, especially no superinfections skin irritation, allergic reactions or local or systemic intolerance. Both study preparations were dermatologically very well tolerated.

## Discussion

Antiviral effects of the special propolis extract preparation GH 2002 applied in this trial have been demonstrated in several open studies aimed on the treatment of herpes labialis [29, 31] and herpes zoster [30], including an examination of the dose and effect [29]. A recent study under blinded conditions examined the effects GH 2002 against herpes labialis in patients included in the early stages of the episode [9]. From this controlled trial arose the question whether the effects of propolis would still be observable if patients with a more progressed stage of the episode were treated. We therefore repeated the study with explicit inclusion of patients in the vesicular phase, and still found a clear superiority over the reference lip cream with 5% aciclovir. The latter was selected because it is currently considered the gold standard of local herpes treatment.

The previous study was considered only single-blinded because the creams were used as such, and there was the theoretical possibility of unblinding of the treating physician due to differences in odor and color of the creams [9]. Although there was no indication in the study that this was in fact the case, we avoided this specific problem in this study by adjusting the odor and color of the comparator aciclovir cream, thus rendering the two preparations undistinguishable and ensuring full blinding. The results of this study confirm the previous observations.

The lack of a placebo control might be considered a weakness in the study design. Apart from the fact that the use of placebo in clinical trial is considered unethical by the authorities in Slovakia and the Czech Republic, the comparator was selected and dosed in accordance with current treatment recommendations. The efficacy and safety of aciclovir 5% cream at the applied dose scheme is not doubted and proven in published studies [3, 6, 32]. Likewise, the duration of the single phases of an untreated episode of Herpes labialis is well known and can also be derived from the clinical trials published with nucleoside analogs. Effects in the duration of the single phases observed in a clinical trial can therefore be rated against the known situation in untreated patients. The efficacy observed in the previous propolis study and in this trial in fact confirm the clinical applicability of aciclovir against lip sores, in accordance with the published controlled trials with nucleoside analogs. With propolis there was still an additional clinical advantage over acyclovir. An advantage of propolis over placebo can therefore be safely assumed. Furthermore, the application of the worst case imputation method for missing values supported the robustness of the efficacy conclusions, as this method would by definition favor the comparator in statistical analyses.

In conclusion, this study confirms the clinical efficacy and safety of application as well as a quick onset of effects of propolis GH 2002 extract lip balm 0.5%, with the clinical usefulness now demonstrated for early [9] and late start of treatment during an episode of herpes labialis. Propolis as an active pharmaceutical ingredient of topical medications for the treatment of herpes labialis may therefore be of special importance for patients for whom the current standard treatment is either not available of not tolerated.
